# Post-Operative Opioid Prescribing Practices and Trends Among Urology Residents in the United States

**DOI:** 10.7759/cureus.12014

**Published:** 2020-12-10

**Authors:** James J Kelley, Sharon Hill, Samuel Deem, Nathan E Hale

**Affiliations:** 1 Urology, Charleston Area Medical Center, Charleston, USA

**Keywords:** prescription opioid, urologic procedures

## Abstract

Introduction: With the opioid epidemic escalating across the country, we sought to evaluate and characterize post-operative opioid prescribing habits and trends among urology residents in the United States.

Methods: Urology residents were sent a 16-question survey regarding opioid prescribing patterns, influencing factors, opioid training experience, and amounts of opioids prescribed for common urologic procedures.

Results: One hundred and four urology residents participated in the survey (75% male and 25% female). Common factors influencing opioid prescribing were standard prescribing practice for certain operations (80%), attending/senior resident preference (62.1%), and immediate post-operative pain (54.7%). Residents reported prescribing more opioids at discharge for open abdominal and robotic procedures (167.9 and 134.2 morphine milligram equivalents, MME, respectively) and lower amounts for outpatient surgeries (39.7 and 55.8 MME for vasectomy and transurethral resections). Only 15.5% of residents utilize any formal algorithm for post-operative opioid prescribing at their institution. Further, 51.6% of residents received no formal education on safe opioid prescribing during residency, and only 42.1% routinely assess patient risk for opioid abuse. Urology residents who received formal opioid training prescribed less opioids on average for common urologic procedures compared to those who had not trained.

Conclusions: This study highlights the importance of increasing resident education on opioid prescribing during residency training, as well as an opportunity for the implementation of standardized post-operative opioid prescribing regimens to help improve trends in urology resident opioid prescribing.

## Introduction

Opioid abuse is an epidemic in the United States, with over 19,000 overdose deaths attributed to prescription opioid pain medications reported in 2017 [1]. Physicians can contribute to this epidemic by regularly utilizing and potentially overprescribing opioid pain medication. In fact, from 1991 to 2013 the number of opioid medication prescriptions by physicians increased from 76 million to 200 million [2].

Prescribing pain medications post-operatively can present a challenge for urologic surgeons, as post-procedural pain is an unavoidable consequence of most surgeries. Urologists must balance the need for sufficient pain control with the potential risks of overprescribing. In a 2011 study, patients who had undergone urologic surgery reported using 58% of the prescribed opioid pain medication, and 67% had excess opioids from their initial prescription [3]. Furthermore, in a study that reviewed patients who had undergone a major prostate or kidney operation, 60% of opioids prescribed post-operatively went unused, resulting in large amounts of excess oxycodone-equivalents in the community [4].

During training, residents are routinely tasked with the responsibility of prescribing pain medication post-operatively. However, post-operative opioid prescribing patterns and the influencing factors on resident prescribing habits are not well-defined in the urological community. To the best of our knowledge, no formal review to date of resident opioid prescribing practices post-operatively has been conducted among residents in urological surgery training programs. The objective of this study was to further assess and characterize post-operative opioid-prescribing habits and trends, influencing factors on prescribing, and formal pain medication prescription training among urology residents.

## Materials and methods

This was a descriptive study performed after obtaining Institutional Review Board approval. An invitation to an online survey was distributed to program coordinators and directors at all Accreditation Council for Graduate Medical Education (ACGME) approved urology residency programs in the United States in October 2018. They were asked to send the survey to all current residents at their respective programs. Surveys were completed electronically using a web-based survey tool (www.surveymonkey.com, SurveyMonkey Inc., San Mateo, CA; a copy of the survey is attached as the Appendix). Survey responses were anonymous, and a link to the survey allowed respondents to complete the survey only once. A total of 104 urology residents responded to the survey and 97 completed the survey. The survey included 16 response items including demographics, post-operative opioid prescribing habits, influencing factors on opioid prescribing, formal education received on opioid and non-opioid adjunctive pain management during residency, assessment for potential patient opioid abuse, and use of formal algorithms for post-operative opioid prescribing. Respondents were asked to report the typical number of pills they would prescribe post-operatively at discharge for eight different common urological procedures including uncomplicated ureteroscopic stone treatment, percutaneous nephrolithotomy, vasectomy, transurethral resection surgery, inflatable penile prosthesis, female mid-urethral sling, robotic surgery, and open abdominal surgery. The amount of opioids administered were converted into morphine milligram equivalents (MME) (the conversion ratio is 1:1.5 for oxycodone to morphine) [5]. Resident responses were compared based on whether they had received formal opioid prescribing education. Responses were compared using paired t-test, and statistical analyses were performed using Statistical Analysis Software (SAS) version 9.3 (SAS Institute Inc., Cary, NC).

## Results

A total of 104 urology residents participated in the survey, with 78 male and 26 female respondents (75% and 25%). The mean age of participants was 29.7 ± SD 2.1. All post-graduate year (PGY) levels were represented with 16 PGY-1 (15.4%), 23 PGY-2 (22.1%), 19 PGY-3 (18.3%), 24 PGY-4 (23.1%), and 22 PGY-5 or greater (21.2%). Residents from 26 different states across the United States responded to the survey. Respondents were from the following geographic areas: 40.21% from the Midwest, 29.9% from the Northeast, 23% from the Southeast, 5% from the Southwest, and 1% from the West. Residents reported race was predominantly Caucasian (87.5%), followed by Asian (4.8%), Hispanic (4.8%), African-American (1%), Middle-Eastern (1%), and American Indian (1%). Table 1 includes the above demographics.

**Table 1:** Demographics of urology resident survey respondents PGY-1 - postgraduate year one; PGY-2 - postgraduate year two; PGY-3- postgraduate year three; PGY-4- postgraduate year four; PGY-5+- postgraduate year five and more.

Demographics of Survey Respondents			
Characteristic		N	%
Sex			
	Male	78	75
	Female	26	25
Race/ethnicity			
	Caucasian	91	87.5
	Asian	5	4.8
	Hispanic	5	4.8
	African Ameican	1	1
	American Indian	1	1
	Middle-Eastern	1	1
Resident level			
	PGY-1	16	15.4
	PGY-2	23	22.1
	PGY-3	19	18.3
	PGY-4	24	23.1
	PGY-5+	22	21.2
Regional variation in response to the survey			
	Midwest	39	40.21
	Northeast	29	29.9
	Southeast	23	23.71
	Southwest	5	5.15
	West	1	1.03

**Figure 1:**
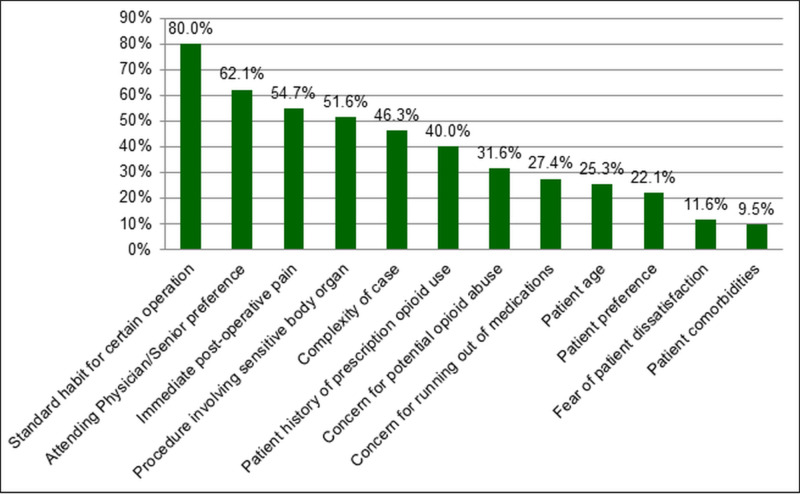
Resident reported factors influencing the type and amount of opioid pain medication prescribed. Residents were able to select up to five influencing factors.

Residents were asked to select the five factors most influencing their opioid prescribing. Responses from most to least influential included (Figure 1): standard prescribing practice for certain operation (80%), attending physician/senior resident preference (62.1%), immediate post-operative pain (54.7%), the procedure involving a more sensitive body organ (51.6%), the complexity of the case (46.3%), patient history of prescription opioid abuse (40%), concern for potential opioid abuse (31.6%), concern for a patient running out of medication (27.4%), patient age (25.3%), patient preference (22.1%), fear of patient dissatisfaction (11.6%), and patient comorbidities (9.5%). When asked to select which of those factors they would consider to be the most influential, responses were similar, with the standard prescribing practice for a certain operation as the highest at 42.1%, followed by attending/senior resident preference (16.8%), immediate post-operative pain (12.6%), the complexity of the case (12.6%), a procedure involving a more sensitive body organ (11.6%), concern for a patient running out of medication (2.1%), and patient comorbidities (1%).

Urology residents commonly prescribe opioid medications at discharge after surgery, with 97% (n=101) of residents reporting that they had prescribed opioids post-operatively within the last year. The most commonly reported types of opioid medications prescribed by residents were hydrocodone-acetaminophen (42.1%), followed by oxycodone (30.5%), oxycodone-acetaminophen (19%), tramadol (6.3%), and codeine/acetaminophen-codeine (1%). The frequency with which patients were instructed to take the opioid prescription (as needed) was every six hours (71.6%), followed by every four hours (24.2%), and eight hours (3.2%).

**Figure 2:**
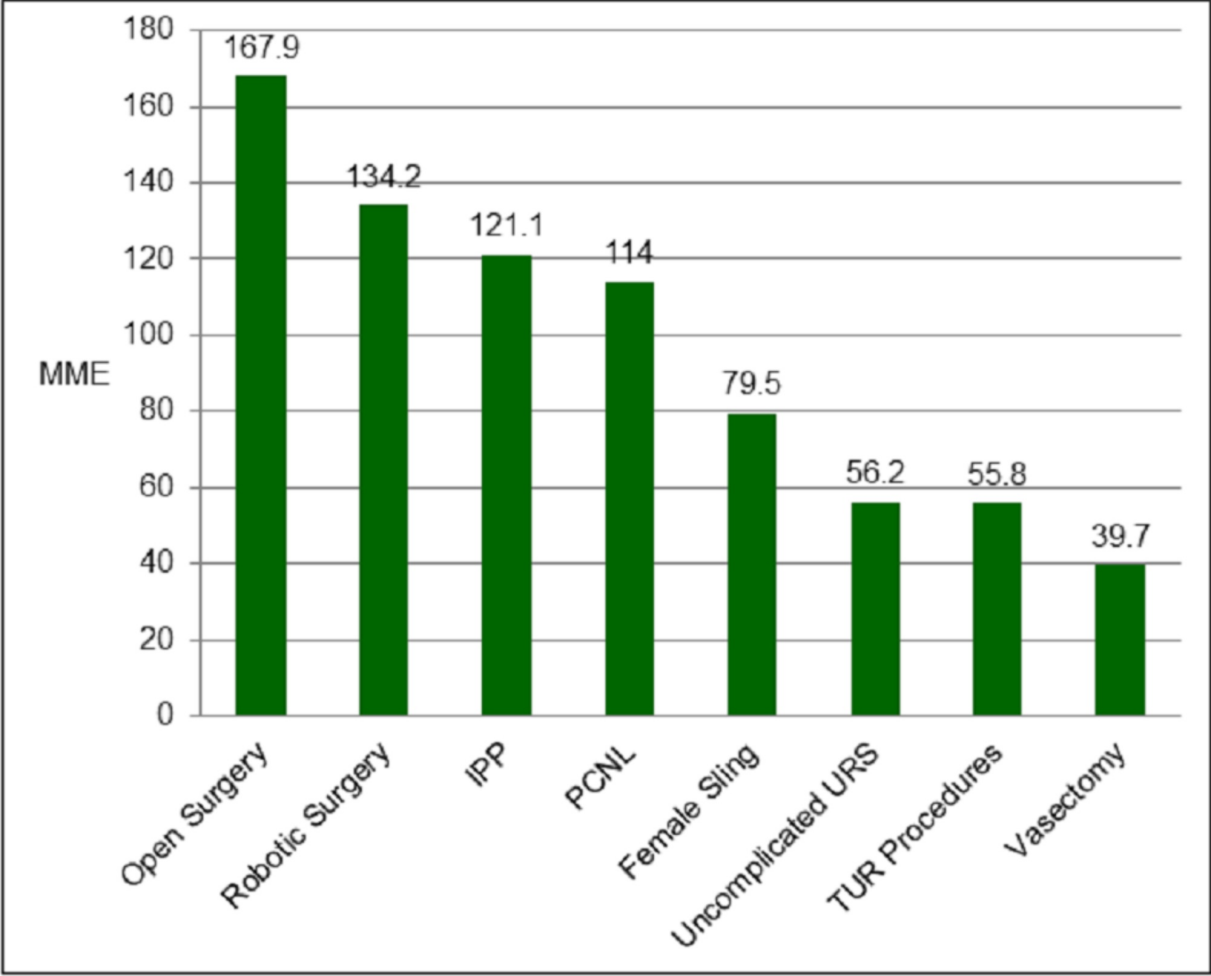
Resident reported MME prescribed post-operatively at discharge for common urologic procedures IPP: inflatable penile prosthesis, PCNL: percutaneous nephrolithotomy, URS: ureteroscopy, TUR: transurethral resection, MME: morphine milligram equivalents.

Residents were surveyed regarding the amount of opioids they would typically prescribe at discharge for eight common urologic procedures. The procedures were uncomplicated ureteroscopic stone treatment, percutaneous nephrolithotomy, vasectomy, transurethral resection surgery, inflatable penile prosthesis, female mid-urethral sling, robotic surgery, and open abdominal surgery. They were asked to report the typical number of pills prescribed when discharging a patient home after the respective surgery based on the assumption that the patient is a 70 kg narcotic-naive adult without comorbidities or any home medications. The prescription would be for oxycodone/acetaminophen 5 mg/325 mg. Residents reported prescribing on average 39.7 MME (5.3 pills) for a vasectomy, 55.8 MME (7.4 pills) for transurethral resection procedures, 56.2 MME (7.5 pills) for uncomplicated ureteroscopic stone treatment, 79.5 MME (10.6 pills) for a female mid-urethral sling, 114 MME (15.2 pills) for percutaneous nephrolithotomy, 121.1 MME (16.1 pills) for inflatable penile prosthesis, 134.2 MME (17.9 pills) for robotic surgeries, and 167.9 MME (22.4 pills) for open abdominal surgeries (Figure 2). 

When asked if their respective residency program utilized any formal method or algorithm for post-operative opioid prescribing, only 15.5% of urology residents responded affirmatively, with the other 84.5% reported not having or being unsure of any existing algorithm at their institution. When asked if they regularly assessed patients’ risk for opioid abuse when prescribing opioid pain medication post-operatively, only 42.1% of residents reported “always” or “often” assessing for risk, with the remaining 58.9% reporting “sometimes,” “rarely,” or “never.” Residents did report routinely prescribing non-opioid adjunctive pain medications at discharge, with 70.5% reporting “always” or “often” prescribing these adjunctive medications. However, no specific information has been requested.

A comparison to locate the difference in prescribing practices between the junior (PGY1-3) and senior (PGY4-5) residents was performed. Senior residents reported less amount of opioids prescription post-operatively than junior residents for most common urological procedures, except for uncomplicated ureteroscopic stone treatment, although the results were not statistically significant (Table 2).

**Table 2:** Comparison of MME prescribing practices between junior (PGY1-3) and senior (PGY4-5) residents MME: morphine milligram equivalents, PGY: post-graduate year, IPP: inflatable penile prosthesis, PCNL: percutaneous nephrolithotomy, URS: ureteroscopy, TUR: transurethral resection, MME: morphine milligram equivalents.

Procedure (mean morphine equivalent)	Senior resident, N=43	Junior resident, N=54	p-Value
URS	60.5 (±39.0)	52.5 (±47.9)	0.3861
PCNL	109.3 (±39.7)	117.9 (±54.7)	0.3944
Vas	39.7 (±40.9)	39.8 (±34.8)	0.9717
TUR	50.7 (±60.7)	60.0 (±54.5)	0.4414
IPP	118.2 (±54.8)	126.2 (±64.6)	0.5323
Sling	69.6 (±50.0)	91.0 (±51.1)	0.0544
Robot	123.5 (±59.9)	143.6 (±69.9)	0.1451
Open	162.0 (±53.9)	173.0 (±76.7)	0.4239

Only 48.4% of residents reported receiving some form of formal instruction or training regarding safe opioid prescribing practices during residency, with the other 51.6% being unsure of or not having received any training. Additionally, when asked if they had received any formal education or training on best practices for pain control during residency, only 34% reported they had received any education, with the remaining 66% reporting “No” or “Unsure."

When comparing residents who had received formal education on safe opioid prescribing to those who had not, those who had received training were more likely to prescribe less opioids post-operatively on average for most common urologic procedures, with the exception of vasectomy and female mid-urethral sling surgeries, although these differences were not found to be statistically significant (Table 3).

**Table 3:** Comparison of MME prescribed by urology residents post-operatively after common urologic procedures depending on if they had received formal education/training on safe opioid prescribing practices or not MME: morphine milligram equivalents.

Procedure	MME if received formal training (n=47)	MME if no formal training (n=50)	p-Value
Uncomplicated ureteroscopic stone treatment	55.8 (±42.3)	56.5 (±46.0)	0.944
Percutaneous nephrolithotomy	108.6 (±49.0)	119.4 (±47.9)	0.286
Vasectomy	41.9 (±38.3)	37.6 (±37.0)	0.598
Transurethral resection	51.3 (±58.8)	60.0 (±56.0)	0.471
Inflatable penile prosthesis	116.2 (±59.8)	128.9 (±60.1)	0.321
Female mid-urethral sling	80.7 (±48.6)	80.2 (±54.5)	0.966
Robotic surgery	125.2 (±59.3)	143.2 (±71.3)	0.193
Open abdominal surgery	162.9 (±62.6)	173.0 (±71.7)	0.473
All procedure means combined	90.4 (±40.5)	101.9 (±45.2)	0.227

Furthermore, urology residents who received formal opioid prescribing education assessed patient risk for opioid abuse “always” or “often” more routinely than those who had not (46.8% vs. 37.5%, p=0.358).

## Discussion

Urology residents are routinely utilizing opioids for pain control post-operatively at discharge for common urologic procedures, with 97% reporting prescribing opioids post-operatively within the last year. While non-opioid regimens have been proposed for several urologic surgeries [6,7], it remains quite common for residents to rely on opioids for post-operative pain control. This is not exclusive to urologic trainees, as it coincides with practices among urologists around the United States, with multiple studies noting routine excessive opioid prescribing after urologic surgery [3,4].

Urology residents appear to be prescribing large amounts of opioids at discharge for several different urologic procedures, in potentially excess quantities. For example, after robotic urologic surgeries, residents reported providing on average 134.2 MME (17.9 pills of oxycodone/acetaminophen 5 mg/325 mg), and for uncomplicated ureteroscopic stone treatment, residents reported 56.2 MME (7.5 pills). This type of excess opioid prescribing can result in large amounts of opioids in the community, as noted in a study by Theisen et al. where 60% of opioids prescribed post-operatively went unused after major prostate or kidney operations [4]. Additionally, studies have shown that non-opioid pathways are feasible after multiple urologic procedures, including ureteroscopy [6] and robotic cystectomy with urinary diversion [7]. As more non-opioid post-operative regimens are described, there may be more opportunities to implement these strategies further in the urologic community.

Furthermore, it is intriguing that most urology residents (80%) reported prescribing standard amounts of opioids for certain operations, yet only 15.5% reported that their respective residency program utilized any formal method or algorithm for post-operative opioid prescribing. Recommendations have been proposed for opioid prescribing after endourologic and minimally invasive urologic procedures [8], and guidelines such as these may be an appropriate starting point for training programs moving forward.

Why urology residents are continuing to prescribe opioids post-operatively is likely multifactorial, as a high percentage (80%) of urology residents in this study reported using a standard prescribing habit post-operatively for certain operations, and 62.1% based their prescribing on their respective attending physician or senior resident preferences. In another survey-based study that evaluated general surgery residents by Chiu et al. [9] residents’ prescribing habits were most commonly influenced by attending physician and senior resident preference (95.2%). These findings would suggest that a major factor in opioid prescribing may be learned practices and behaviors of trainees, which again suggests a culture of overprescribing in the surgical communities.

There also appears to be a great opportunity for urology resident education on opioid prescribing practices. Less than half (48.4%) of urology residents reported receiving any form of formal instruction or training regarding safe opioid prescribing practices during residency, and only 34% reported any formal education/training on best practices for pain control. This lack of opioid education during residency has been described in other surgical fields, as reported by Chiu et al. with only 6% of general surgery residents who were surveyed reporting any formal training in opioid prescribing, and 8.5% receiving formal training in best practices of pain management [9]. Educational programs on pain management during residency training have been found previously to be effective [10]. In fact, in our study, it was noted that when comparing urology residents who had received formal opioid prescribing training during residency to those who had not, those who received training on average prescribed less MME (101.9 vs. 90.4). These data highlight the importance of further opioid education of urology trainees in the future.

While this study is the first to our knowledge to investigate opioid prescribing practices among residents in urology training programs and illustrates several interesting resident practices and trends, it is not without limitations. First, our sample size included 104 survey respondents, which is roughly 8% of total current residents in ACGME accredited urology programs [11]. It is notable, however, that the demographic distributions of residents were similar overall to urology programs as a whole [11]. Second, although several overall trends in the sub-analysis comparison of residents were noted, no statistically significant differences were appreciated, and we believe that this is likely attributable to sample size. Finally, this was a self-reported survey, and specific prescribing amounts/patterns were not assessed, which may allow for the potential of reporting and recall bias.

## Conclusions

Urology residents commonly prescribe opioids for post-operative pain control, yet over half of residents receive no formal education or training on safe opioid prescribing practices. The majority of residents routinely give a standard amount of opioids to all patients for a certain procedure, yet few reports having any formal algorithm for post-operative opioid-prescribing at their institution. Residents who received safe opioid education prescribed less opioids on average at discharge and assessed patient risk for opioid abuse more often. This study highlights the importance of increasing resident education on opioid prescribing during residency training, as well as illustrates an opportunity for implementation of standardized post-operative opioid prescribing regimens to help improve trends in urology resident opioid prescribing and allow urology residents to play a more substantial role in combatting the opioid epidemic.

## Appendices

Urology Resident Survey

1.       What is your sex?

a.       Male

b.      Female

2.       How would you describe yourself?

a.       White/Caucasian

b.      Black/African American

c.       Hispanic/Latino

d.      American Indian or Alaska Native

e.       Asian

f.        Native Hawaiian or Pacific Islander

g.       Other (please specify)

3.       What is your age?

a.       Drop down selection (1-99)

4.       What is your current post-graduate year in training?

a.      PGY-1

b.      PGY-2

c.      PGY-3

d.      PGY-4

e.      PGY-5 or greater

5.       What state are you completing your current residency in?

a.       Drop down selection (All 50 United States)

6.       In the last year, have you prescribed opioid pain medications for post-operative patients upon discharge following a urological procedure?

a.       Yes                                                             i.     If Yes, proceed to next line of questions

b.       No                                                              i.     If No, skips to question 12

7.       Which opioid pain medication would you say you most commonly prescribe when giving a post-operative opioid pain prescription?

a.      Hydrocodone-acetaminophen (Norco/Lortab)

b.      Hydrocodone

c.      Oxycodone-acetaminophen (Percocet)

d.      Oxycodone

e.      Hydromorphone (Dilaudid)

f.       Codeine/Acetaminophen-codeine

g.      Tramadol

h.      Other

8.       With what frequency do you recommend that a patient take their opioid pain medication?

a.      q 2 hr

b.      q 4 hr

c.      q 6 hr

d.      q 8 hr

e.      q 12 hr

f.       Other (please specify)

9.       For each of these common urologic procedures listed below, what number of pills would you typically prescribe for post-operative pain utilizing oxycodone-acetaminophen 5-325 mg (Percocet 5-325 mg)? Consider these patients to be 70 kg, narcotic-naive adults, without comorbidities or any home medications. This is the prescription you would write to them when they are going home.

a.      Uncomplicated ureteroscopy with stone treatment

b.      Percutaneous nephrolithotomy

c.       Vasectomy

d.      Transurethral resection surgery (TURP, TURBT, etc.)

e.       Inflatable penile prosthesis placement

f.        Female mid-urethral sling placement

g.       Robotic surgery (prostatectomy, partial/radical nephrectomy, sacrocolpopexy, etc.)

h.      Open abdominal surgery (nephrectomy, cystectomy, urinary diversion, etc.)

10.   Which of the following factors most influences the type and amount of opioid pain medication you prescribe? (May choose up to 5)

a.      Standard prescribing habit for all patients of a certain operation

b.      Attending Physician/Senior resident preference

c.      Patient preference

d.      Patient history of prescription opioid use

e.      Patient comorbidities

f.       Patient age

g.      Concern for potential opioid abuse

h.      The complexity of the case

i.       A procedure involving a more sensitive body organ (i.e. scrotum, penis, vagina)

j.       Immediate post-operative pain

k.      Concern for the patient running out of medications

l.       Fear of patient dissatisfaction

m.     Other (please specify)

11.   Which of the following factors would you consider to be the most influential on the type and amount of opioid pain medication you prescribe? (May choose more than one)

a.      Standard prescribing habit for all patients of a certain operation

b.      Attending Physician/Senior resident preference

c.      Patient preference

d.      Patient history of prescription opioid use

e.      Patient comorbidities

f.       Patient age

g.      Concern for potential opioid abuse

h.      Complexity of the case

i.       Procedure involving a more sensitive body organ (i.e. scrotum, penis, vagina)

j.       Immediate post-operative pain

k.      Concern for the patient running out of medications

l.       Fear of patient dissatisfaction

m.     Other (please specify)

12.   How often do you assess your patient’s risk for potential opioid abuse prior to prescribing?

a.      Always

b.      Often

c.      Sometimes

d.      Rarely

e.      Never

13.   How often do you prescribe adjunct non-opioid pain medications for post-operative patients upon discharge from the hospital?

a.      Always

b.      Often

c.      Sometimes

d.      Rarely

e.      Never

14.   Do you have a formal method or algorithm for prescribing post-operative opioid pain medications at your program?

a.      Yes

b.      No

c.      Unsure

15.   Have you received formal instruction and/or training on safe opioid pain medication prescribing practices during residency?

a.      Yes

b.      No

c.      Unsure

16.   Have you had formal instruction and/or training on best practices for pain control?

a.      Yes

b.      No

c.      Unsure

## References

[1] (2019). Overdose death rates. National Institute on Drug Abuse, 29 Jan.

[2] Volkow Volkow, N N (2019). America's addiction to opioids: heroin and prescription drug abuse. Senate Caucus on International Narcotics Control, 14 May.

[3] Bates C, Laciak R, Southwick A, Bishoff J (2011). Overprescription of postoperative narcotics: a look at postoperative pain medication delivery, consumption and disposal in urological practice. J Urol.

[4] Theisen K, Myrga J, Hale N (2019). Excessive opioid prescribing after major urologic procedures. Urology.

[5] Gordon DB, Stevenson KK, Griffie J, Muchka S, Rapp C, Ford-Roberts K (2005). Opioid equianalgesic calculations. J Palliat Med.

[6] Sobel DW, Cisu T, Barclay T, Pham A, Callas P, Sternberg K (2018). A retrospective review demonstrating the feasibility of discharging patients without opioids after ureteroscopy and ureteral stent placement. J Endourol.

[7] Audenet F, Atalla K, Giordano M (2019). Prospective implementation of a nonopioid protocol for patients undergoing robotic-assisted radical cystectomy with extracorporeal urinary diversion. Urol Oncol.

[8] Koo K, Faisal F, Gupta N (2020). Recommendations for opioid prescribing after endourological and minimally invasive urological surgery: an expert panel consensus. J Urol.

[9] Chiu AS, Healy JM, DeWane MP, Longo WE, Yoo PS (2018). Trainees as agents of change in the opioid epidemic: optimizing the opioid prescription practices of surgical residents. J Surg Educ.

[10] Scott E, Borate U, Heitner S, Chaitowitz M, Tester W, Eiger G (2008 Sept). Pain management practices by internal medicine residents--a comparison before and after educational and institutional interventions. Am J Hosp Palliat Care.

[11] (2019). Urology residency match statistics. American Urological Association, 17 Jan.

